# Development and validation of dual-time-window readmission prediction models under China’s medical insurance multi-payment reform

**DOI:** 10.3389/fpubh.2026.1909323

**Published:** 2026-08-19

**Authors:** Ying Guan, Xianhao Chen, Ting Zhang, Liyu Zhao, Ru Meng, Fengqi Tang, Zaixiang Tan, Xinru Huang

**Affiliations:** 1 School of Management, Xuzhou Medical University, Xuzhou, China; 2 School of Medical Imaging, Xuzhou Medical University, Xuzhou, China; 3 Outpatient Department, Huai’an No.3 People’s Hospital, Huai’an, China; 4 School of Public Health, Xuzhou Medical University, Xuzhou, China; 5 Medical Insurance Department, Xuzhou Cancer Hospital, Xuzhou, China; 6 Institution of Chinese Health Modernization, Xuzhou Medical University, Xuzhou, China; 7 The Affiliated Hospital of Xuzhou Medical University, Xuzhou, China

**Keywords:** 15-day readmission, 30-day readmission, coordinated tripartite medical reform, dual-time-window readmission, logistic regression, multi-payment reform

## Abstract

**Background:**

Readmission is an important indicator of healthcare quality, reflecting continuity of care, treatment outcomes, and the quality of discharge management. However, existing readmission prediction studies have primarily focused on 30-day readmission as the primary outcome, with limited attention to differences in the underlying risk mechanisms, predictive performance, and clinical applicability across different readmission time windows. Meanwhile, under the context of China’s multi-payment reform and the Coordinated Tripartite Medical Reform, limited evidence is available on the development of readmission prediction models based on real-world data and their application to healthcare quality monitoring and the management of high-risk patients. Therefore, this study aimed to develop and validate 15-day and 30-day all-cause same-hospital readmission prediction models using real-world hospitalization data, compare the predictive factors and model performance across different readmission time windows, and provide empirical evidence to support healthcare quality monitoring and risk management under the ongoing payment reform.

**Methods:**

A single-center retrospective design was adopted, and data from 57,677 hospitalizations at a large tertiary hospital in China between January 2021 and July 2025 were collected. Multivariable logistic regression models were developed for 15-day and 30-day all-cause same-hospital readmissions, respectively. Model performance was evaluated using the AUC, Brier score, calibration plots, bootstrap internal validation, and temporal external validation. Differences in predictor associations between the two readmission time horizons were further examined.

**Results:**

The 15-day and 30-day readmission rates were 9.47 and 10.92%, respectively. The 15-day model achieved an AUC of 0.840 (95% CI: 0.828–0.843) and a Brier score of 0.0620; the 30-day model achieved an AUC of 0.820 (95% CI: 0.810–0.825) and a Brier score of 0.0735. Both AUCs were statistically significant. Both models demonstrated good discrimination and calibration, with the 15-day model consistently outperforming the 30-day model across all performance metrics. In the temporal external validation, the model achieved an AUC of 0.810 and a Brier score of 0.136, indicating good generalizability. Age, LOS, surgical status, and number of secondary diagnoses were independently associated with higher odds of readmission (overall *p* < 0.05), while higher total cost was associated with lower odds of readmission. Risk stratification showed that the observed readmission rate exceeded 58% in the highest-risk decile and was below 2% in the lowest-risk group.

**Conclusion:**

This study developed and validated dual-time-horizon readmission prediction models based on real-world data, extending the conventional approach of single-horizon readmission risk assessment. The models provide a methodological framework for risk stratification and healthcare quality monitoring across different post-discharge periods. They may support early discharge risk screening, facilitate prioritization of transitional care resources for high-risk patients, and provide empirical evidence to inform quality assessment, risk adjustment, and management decision-making under the multi-payment reform and the Coordinated Tripartite Medical Reform.

## 1 Introduction

Public health systems are transitioning from fragmented service delivery toward systematic integration (1, 2). Coordination of healthcare delivery, health insurance payment management, and pharmaceutical resource allocation has become a central issue in global health policy (3). In this context, China has proposed the Coordinated Tripartite Medical Reform, which integrates pharmaceuticals, healthcare services, and health insurance to strengthen coordination across the healthcare system. The reform aims to optimize healthcare delivery through payment reform, improve resource allocation and quality management, and achieve a balance between cost containment, healthcare quality improvement, and better health outcomes (4). Among these components, health insurance payment mechanisms represent a key policy instrument linking healthcare delivery with resource allocation. By influencing provider incentives, payment reforms can reshape hospital operations and promote healthcare quality management.

Within the context of the Coordinated Tripartite Medical Reform, the multi-payment reform has become an important direction for transforming China’s health insurance payment system. The multi-payment reform refers to the transition from a single payment approach toward a diversified payment framework that integrates multiple payment methods according to disease characteristics, clinical practice patterns, resource consumption, and service needs. These methods include the Diagnosis-Intervention Packet (DIP) payment, the Diagnosis-Related Group (DRG) payment, the per diem payment, and the capitation payment. By combining the advantages of different payment approaches and implementing tailored payment strategies, the reform aims to achieve cost containment, quality improvement, efficiency enhancement, and more rational allocation of healthcare resources (5). Within this framework, different payment methods serve distinct policy functions. The DIP payment system determines the payment value based on regional case-mix characteristics and resource consumption patterns, aiming to improve the efficiency of health insurance fund utilization by encouraging healthcare providers to optimize treatment practices, standardize clinical pathways, and control unnecessary expenditures (6). For psychiatric care, rehabilitation services, and other conditions requiring prolonged hospitalization, the per diem payment is still adopted in many regions as a complementary approach due to the specific characteristics of these conditions and their long-term care needs (7). Therefore, the health insurance payment reforms implemented in some regions of China represent the development of a multi-payment system rather than a transition toward a single payment model. However, while different payment methods may promote efficient resource allocation and cost control, they may also generate heterogeneous effects on healthcare quality. For example, the DIP payment may encourage hospitals to shorten the length of stay and improve service efficiency, but insufficient quality monitoring mechanisms may increase the risk of premature discharge and unplanned readmission (8). Although the per diem payment can ensure continuous care for patients requiring long-term hospitalization, the fixed daily reimbursement structure may weaken incentives for hospitals to optimize care processes and improve service quality (9). Therefore, under the multi-payment reform framework, establishing effective quality monitoring mechanisms that balance cost containment and healthcare quality improvement has become an important challenge for advancing health insurance payment reform and achieving the coordinated governance of healthcare services.

Unplanned readmission within 30 days is widely recognized as a core indicator of care quality, continuity of care, and system efficiency (10, 11). Avoidable readmissions increase patients’ physical and psychological burdens and medical expenditures also constitute inefficient use of health insurance funds. Readmission rates reflect the functioning of three interconnected systems. First, they capture the quality of inpatient care, the prudence of discharge decisions, and access to transitional care (12). Second, they represent additional costs borne by payers, which is why many health insurance systems have incorporated readmission into penalty-based or incentive-based payment mechanisms (13, 14). Third, they are related to the appropriateness of discharge medications (15), adverse drug reactions (16), and medication adherence—all common correlates of early readmission. However, the appropriate time window for assessing readmission remains under debate, although readmission is widely recognized as an important indicator of healthcare quality. Previous studies have commonly used 30-day readmission as the key measure for quality evaluation and risk prediction. Increasing attention has been paid to whether readmissions occurring within different time windows reflect distinct clinical meanings and predictive values. Previous evidence suggests that early readmissions are more closely associated with transitional care after discharge, continuity of treatment, and potentially modifiable healthcare-related factors. In contrast, later readmissions may be more strongly influenced by disease progression, long-term health conditions, and social determinants (17). These findings suggest that different readmission windows may capture different aspects of healthcare quality. Shorter time windows may be more sensitive to risk signals related to inpatient care quality and discharge management, although further validation is still required. In addition, the selection of prediction windows may influence the identification of risk factors, model performance, and clinical applicability. Henderson et al. compared readmission prediction models across different time horizons in more than two million hospitalized patients. Their findings showed that prediction windows affected model discrimination and practical application scenarios, suggesting that different time windows may serve different objectives in healthcare quality management (18). Therefore, under the context of multi-payment reform, evaluating the performance of readmission risk prediction models across different time windows may provide important evidence for developing more accurate and effective healthcare quality monitoring systems.

Similar frameworks have been developed internationally. The Institute for Healthcare Improvement in the United States proposed the Triple Aim, which advocates simultaneous improvement in population health, patient experience, and per capita cost reduction (19). Building on this framework, value-based care further links payment to clinical outcomes. Practices such as Germany’s Gesundes Kinzigtal program (20) and integrated care systems in the United Kingdom (21) indicate that payment reform should be accompanied by service integration and quality monitoring, with readmission rates serving as a key cross-system indicator. In recent years, researchers in China and other countries have developed numerous readmission prediction models using real-world data. Logistic regression models based on Diagnosis-Related Groups (DRG-based) data have been used to assess hospital readmission risk, and their findings have revealed the complex role of LOS across different analytic levels (8). Machine learning methods have also been applied to readmission prediction, with feature-importance analyses identifying age, LOS, specific surgical procedures, and discharge against medical advice as key predictors (22). Existing studies on health insurance payment reform in China have mainly focused on economic and efficiency-related outcomes, including cost containment, healthcare utilization, and changes in provider behavior. However, limited attention has been paid to healthcare quality outcomes under the context of payment reform. Readmission, as an important indicator reflecting healthcare continuity, treatment effectiveness, and discharge management quality, provides valuable evidence for quality monitoring under the multi-payment reform framework. Although readmission prediction models have been increasingly developed in recent years, several limitations remain. First, the current multi-payment reform is still primarily oriented toward cost control, while the integration of healthcare quality outcomes into the payment system remains insufficient. Second, existing readmission prediction studies have predominantly focused on 30-day readmission, whereas differences in risk mechanisms and predictive performance across different readmission periods have received limited attention. Kansagara et al. conducted a systematic review and found that most existing readmission prediction models focused on 30-day readmission, with insufficient reporting on model validation and calibration (23). Henderson et al. further demonstrated that prediction models based on different time windows provide complementary predictive value, and models using shorter time windows may achieve better predictive performance than those using longer time windows (18). However, under the context of multi-payment reform, studies that simultaneously develop and compare readmission prediction models across different time windows remain limited.

To address these gaps, this study used real-world hospitalization data from 57,677 patients admitted to a large tertiary hospital in China between January 2021 and July 2025. Multivariable logistic regression models were developed for 15-day and 30-day all-cause same-hospital readmissions, respectively. Logistic regression was used, and age, LOS, surgical status, number of secondary diagnoses, primary diagnosis code, and discharge year were included as predictors. Model performance was comprehensively evaluated using the AUC, Brier score, calibration plots, and bootstrap internal validation. This study aimed to construct and evaluate 15-day and 30-day readmission prediction models within the multi-payment reform, characterize associations of predictor variables with readmission across time windows, and interpret the policy implications of the findings under the coordinated tripartite medical reform framework. The findings are intended to provide empirical evidence for linking quality monitoring with payment mechanisms in multi-payment reform.

## 2 Methods

### 2.1 Study design and data source

A single-center retrospective study design was used. Data were obtained from electronic medical records and health insurance settlement records of inpatients admitted to a large tertiary hospital in China from January 2021 to July 2025. A total of 57,677 hospitalization records were included, each representing one complete hospitalization episode. The data fields included age, sex, LOS, total hospitalization cost, number of operations, number of secondary diagnoses, primary diagnosis code, discharge year, and readmission status. All data were de-identified after extraction to ensure patient privacy protection.

### 2.2 Ethics statement

Retrospective, fully de-identified administrative data were used in this study, and no personal identifiable information was involved. According to the policy of the institutional ethics committee, analyses of anonymized secondary administrative data were exempt from formal ethics review. Therefore, ethics approval and informed consent were not required.

### 2.3 Data preprocessing

A total of 58,711 hospitalization cases from January 2021 to July 2025 were initially identified. To ensure data quality and model stability, the following preprocessing steps were applied to the raw data.

(1) Follow-up time was truncated. To ensure complete follow-up for both the 15-day and 30-day readmission outcomes, all discharge cases occurring after July 2025 were excluded (*n* = 1,017), because 30-day follow-up could not be completed for these patients. For the 15-day readmission outcome, at least 15 days of potential follow-up were also required for each patient; therefore, the same exclusion criterion was applied to both outcomes. A final set of 57,694 hospitalization cases was retained for descriptive statistics and estimation of overall readmission rates.
(2) Missing values were handled using complete-case analysis for multivariable modeling. Records with missing core predictors, including age, LOS, total cost, number of operations, number of secondary diagnoses, primary diagnosis code, and discharge year, were removed. The proportion of missing data was less than 0.1% (17/57,694) and was considered negligible. Given this very low level of missingness, complete-case analysis was unlikely to materially alter model estimates.

### 2.4 Outcome definition

The primary outcomes of this study were 15-day and 30-day all-cause same-hospital readmission. All-cause readmission was defined as any hospitalization occurring within 15 or 30 days after discharge from the index hospitalization, regardless of the reason, in the same hospital. Unplanned readmission is commonly used as an indicator for evaluating healthcare quality and refers to re-hospitalization caused by disease progression, treatment-related problems, or other unexpected factors. In contrast, all-cause readmission includes both planned and unplanned readmission events. Since the administrative hospitalization data used in this study lacked reliable information to identify whether readmissions were planned or unplanned, these two types of readmission could not be accurately distinguished. Therefore, all same-hospital readmission events were included in the analysis, and the outcomes were defined as all-cause same-hospital readmission rather than strictly unplanned readmission (23, 24).

### 2.5 Variable definitions and filtering

1 Age was treated as a continuous variable over the years and was defined as the patient’s exact age at admission.
2 Clinical variables were defined as follows.

  (1) LOS was treated as a continuous variable in days and was calculated as the number of days from admission to discharge.
  (2) Surgical status was treated as a binary variable and was categorized according to whether surgery was performed during hospitalization: no surgery (reference group) and surgery. A binary rather than multicategory specification was used because the presence or absence of surgery has clear clinical relevance to readmission risk, and sample size assessment indicated that the binary specification provided more stable statistical power.
  (3) The number of secondary diagnoses was treated as a continuous variable and referred to the number of diagnoses or comorbidities other than the principal diagnosis, reflecting the patient’s comorbidity burden.

3 Screening process

Regarding variable selection, this study did not use the results of univariable analyses as the basis for predictor selection. Candidate predictors were identified by considering previous research evidence, clinical relevance, and their potential contributions to readmission risk prediction. The variables ultimately included in the model consisted of patient demographic characteristics, previous hospitalization history, clinical diagnostic information, and payment-related characteristics. For disease-related variables, the primary diagnosis codes were standardized based on the International Classification of Diseases (ICD) coding system. The first three digits of the primary diagnosis codes were extracted to define disease categories. Considering that some diagnosis-code groups contained relatively few cases, which could lead to unstable model estimation, rare code groups with fewer than 50 hospitalization cases were combined into an “Other” category. The resulting categorical variables were then transformed using one-hot encoding. The diagnosis-code group with the largest number of cases was selected as the reference group, and all remaining categories, together with other candidate predictors, were simultaneously entered into the multi-variable logistic regression model for analysis.

### 2.6 Descriptive analysis

Baseline characteristics of the study population were first summarized. Continuous variables were described using medians and interquartile ranges (IQR) given their skewed distributions. Categorical variables were described using frequencies and percentages. Between-group comparisons were performed using the Mann–Whitney U test for continuous variables and the chi-square test for categorical variables.

A total of 57,677 hospitalization records were included for model development. The overall 15-day readmission rate was 9.47%, and the 30-day readmission rate was 10.92%. Baseline characteristics stratified by 15-day readmission status are presented in Table 1, and baseline characteristics stratified by 30-day readmission status are summarized in Table 2.

**Table 1:** Baseline characteristics of the study population stratified by 15-day readmission status.

Variable	15-day non- readmission	15-day readmission	*p*-value
Sample size, n	52,213	5,464	–
Age (years), IQR	50 (30–62)	49 (34–56)	<0.0001
Length of stay (days), IQR	20 (11–32)	91 (29–92)	<0.0001
Total cost (CNY), IQR	11,591 (6,971-20,456)	33,317 (19,875-37,004)	<0.0001
Surgery rate (%)	52.43%	75.70%	<0.0001

**Table 2:** Baseline characteristics of the study population stratified by 30-day readmission status.

Variable	30-day non- readmission	30-day readmission	*P*-value
Sample size, *n*	51,380	6,297	–
Age (years), IQR	49 (30–62)	50 (34–57)	0.0512
Length of stay (days), IQR	20 (11–32)	90 (21–91)	<0.0001
Total cost (CNY), IQR	11,581 (6,956-20,491)	32,309 (12,877-36,452)	<0.0001
Surgery rate (%)	51.98%	76.34%	<0.0001

*p*-values calculated using the Mann–Whitney U test. Values are presented as median (IQR) for continuous variables and n (%) for categorical variables. Total cost is expressed in Chinese Yuan (CNY).

Among patients stratified by 15-day readmission status (Table 1), those who were readmitted had a longer LOS (IQR, 91 vs. 20 days), higher total cost (IQR, 33,317 vs. 11,591 CNY [Chinese Yuan]), and a higher proportion undergoing surgery (75.70% vs. 52.43%) than those who were not readmitted. As shown in Table 2, among patients stratified by 30-day readmission status, patients who were readmitted had a longer LOS (IQR, 90 vs. 20 days), higher total cost (IQR, 32,309 vs. 11,581 CNY), and a higher proportion undergoing surgery (76.34% vs. 51.98%) than those who were not readmitted.

### 2.7 Multicollinearity diagnosis and handling

In the context of multi-payment reform, total hospitalization cost is intrinsically correlated with LOS and primary diagnosis code, because primary diagnosis code is generated from historical cost clustering and LOS is a major driver of cost. Simultaneous inclusion of untransformed cost, length of stay, and primary diagnosis code may introduce multicollinearity, which could compromise the stability of coefficient estimates. To identify potential collinearity, variance inflation factor (VIF) diagnostics were conducted for all continuous predictors before model fitting. When raw total cost (unit: CNY) was used, the VIF values for LOS and total cost were 11.69 and 15.57 (Table 3), respectively, both exceeding the conventional threshold of 10, consistent with strong collinearity. The VIF values for age, surgery status, and number of secondary diagnoses remained within acceptable limits. To mitigate collinearity, natural logarithmic transformation was applied to the total cost variable. After transformation, marginally exceeding the commonly adopted threshold of 10. However, several considerations collectively supported the assessment that this degree of collinearity remained within an acceptable range. First, O’Brien indicated that the VIF threshold of 10 was recommended primarily for small-sample studies (*n* < 200) and could be reasonably relaxed in large-sample contexts (*n* > 200) (25). With 57,677 cases, the present study’s sample size far exceeded this cutoff. Second, the correlation between LOS and total cost did not originate from data collection or measurement error but arose from the intrinsic structural logic of multi-payment reform grouping—namely, that the case scores themselves were generated through clustering of historical costs (26). This structural collinearity reflected an inherent feature of the payment system design rather than a data quality deficiency. Third, the core objective of this study was to construct a risk assessment tool with robust predictive performance, not to perform rigorous causal inference. In a predictive modeling framework, mild instability of coefficient estimates does not substantially compromise overall predictive capacity. In summary, although the VIF for log-transformed total cost slightly exceeded 10, it was notably reduced from 15.57 in the original-cost model, and the VIF values of all remaining variables stayed below 10. This study further conducted the sensitivity analysis using the ridge regression model. The model performance remained stable, with an AUC of 0.8089 and a Brier score of 0.1365 in the validation set, which were comparable to the results of the primary model. These findings indicate that the model was not substantially affected by multicollinearity among predictors.

**Table 3:** Results of multicollinearity diagnosis and handling.

Variable	Original cost model VIF	VIF after logarithmic transformation
Age	3.61	8.86
Length of stay (days)	11.69	2.03
Total cost	15.57	10.44
Surgery	2.26	2.36
Number of secondary diagnoses	2.43	2.50

To further examine the robustness of model performance with respect to alternative cost variable specifications, a sensitivity analysis was conducted comparing two approaches: natural-log transformation of total cost and rescaling of total cost by dividing by 1,000 (unit: CNY). The AUC, Brier score, and direction and statistical significance of the ORs for the core variables were broadly consistent between the two approaches; the AUC of the 15-day model under the log-transformed was 0.840. Given the comparable performance of the two approaches and the more intuitive clinical interpretability of the log transformation, this approach was adopted as the cost variable specification for the primary model.

### 2.8 Model construction

Multivariable logistic regression models were constructed separately for 15-day and 30-day readmission. Logistic regression is a classification method widely used in medical research and is suitable for modeling binary outcome variables. Unlike linear regression, logistic regression maps the linear combination of predictors to the (0,1) interval through a link function, such that the output can be interpreted as the probability of the event. Specifically, the model can be expressed as Equation 1:


$$\log(\frac{p}{1-p})=\beta_{0}+\beta_{1}X_{1}+\beta_{2}X_{2}+\cdots \beta_{k}X_{k}$$ (1)

where p denotes the probability of readmission, X₁, X₂, …, Xk are the predictor variables, β₀ is the intercept, and β₁, β₂, …, βk are the regression coefficients for each variable. The regression coefficients were estimated using maximum likelihood estimation — that is, the parameter estimates that maximize the likelihood function were identified given the observed data. Compared with ordinary least squares estimation, maximum likelihood estimation possesses more favorable statistical properties for binary outcome variables. The exponentiated regression coefficients yield the adjusted odds ratio (OR), which represents the multiplicative change in the odds of readmission associated with a one-unit increase in ecah predictor, with all other variables held constant. The OR and its 95% confidence interval were reported for each variable to assess the direction and strength of the association between each predictor and readmission risk.

### 2.9 Model performance evaluation and internal validation

Model performance for the 15-day and 30-day readmission prediction models was assessed in terms of discrimination, calibration, and overall accuracy, and internal validation was conducted using bootstrap resampling (1,000 iterations).

Discrimination was quantified using AUC, which ranges from 0.5 to 1.0, with values above 0.8 considered indicative of good discrimination. The optimal probability threshold was derived from the Youden index, and sensitivity and specificity at this threshold were reported. Calibration was assessed using decile-based calibration plots, and the Brier score was reported as a measure of overall accuracy. A Brier score below 0.10 was considered indicative of good overall accuracy. Given the large sample size of the present study, traditional goodness-of-fit tests may be overly sensitive to minor deviations; therefore, calibration was primarily judged through visual inspection of the calibration plots. To prevent overestimation of performance attributable to overfitting, internal validation was performed using bootstrap resampling. A total of 1,000 bootstrap samples were drawn with replacement from the original dataset. The model was refitted on each bootstrap sample, and the optimism-corrected AUC was calculated, with optimism defined as the difference between the apparent AUC and the mean bootstrap AUC. This procedure was used to evaluate the generalizability of the model.

All statistical analyses were performed using SPSS 26.0 and Python 3.10, with two-sided *p* < 0.05 considered statistically significant.

## 3 Results

### 3.1 15-day readmission prediction model

A total of 57,677 hospitalization records were included, and the 15-day readmission rate was 9.47% (5,464/57,677). Results from the multivariable logistic regression model are presented in Table 4. After adjusting for age, LOS, total cost, surgery status, number of secondary diagnoses, primary diagnosis code, and discharge year, LOS, age, and number of secondary diagnoses remained independently associated with 15-day readmission (Table 4). Surgery was significantly associated with higher odds of 15-day readmission, whereas Log-transformed total cost was linked to a reduction in readmission.

**Table 4:** Multivariable logistic regression for 15-day readmission.

Variable	OR	95% CI	*P*-value
Age (per year)	1.006	1.004–1.009	<0.001
Length of stay (per day)	1.020	1.019–1.021	<0.001
Total cost (per log unit)	0.619	0.580–0.660	<0.001
Surgery (reference: no surgery)	1.447	1.322–1.583	<0.001
Number of secondary diagnoses (per unit)	1.030	1.010–1.051	0.004
Year of discharge (reference: 2021)			
2022	2.430	1.996–2.958	<0.001
2023	15.338	12.782–18.403	<0.001
2024	16.034	13.310–19.315	<0.001
2025	6.675	5.468–8.148	<0.001
Primary diagnosis code	–	–	<0.001

Each 1-year increase in age corresponded to a 0.62% increase in the odds of readmission (OR = 1.006; 95% CI: 1.004–1.009; *p* < 0.001). Each additional day of LOS was associated with a 1.99% increase in 15-day readmission odds (OR = 1.020; 95% CI: 1.019–1.021; p < 0.001). A 1-unit increase in the natural logarithm of total cost (corresponding to a 2.72-fold increase in cost) was associated with a 38.11% reduction in readmission odds (OR = 0.619; 95% CI: 0.580–0.660; *p* < 0.001). Surgery showed significantly higher odds of 15-day readmission (OR = 1.447; 95% CI: 1.322–1.583; *p* < 0.001). Each additional secondary diagnosis increased the odds by 3.04% (OR = 1.030; 95% CI: 1.010–1.051; *p* = 0.004).

With 2021 as the reference year, the odds of 15-day readmission were significantly elevated in 2022 (OR = 2.430; 95% CI: 1.996–2.958; *p* < 0.001), rose sharply to a peak in 2023 (OR = 15.338; 95% CI: 12.782–18.403; p < 0.001) and 2024 (OR = 16.034; 95% CI: 13.310–19.315; p < 0.001), and declined but remained elevated in 2025 (OR = 6.675; 95% CI: 5.468–8.148; p < 0.001).

As an overall categorical predictor, primary diagnosis code was significantly associated with readmission odds (overall p < 0.001), reflecting substantial heterogeneity in readmission risk across primary diagnosis code.

**Figure 1:**
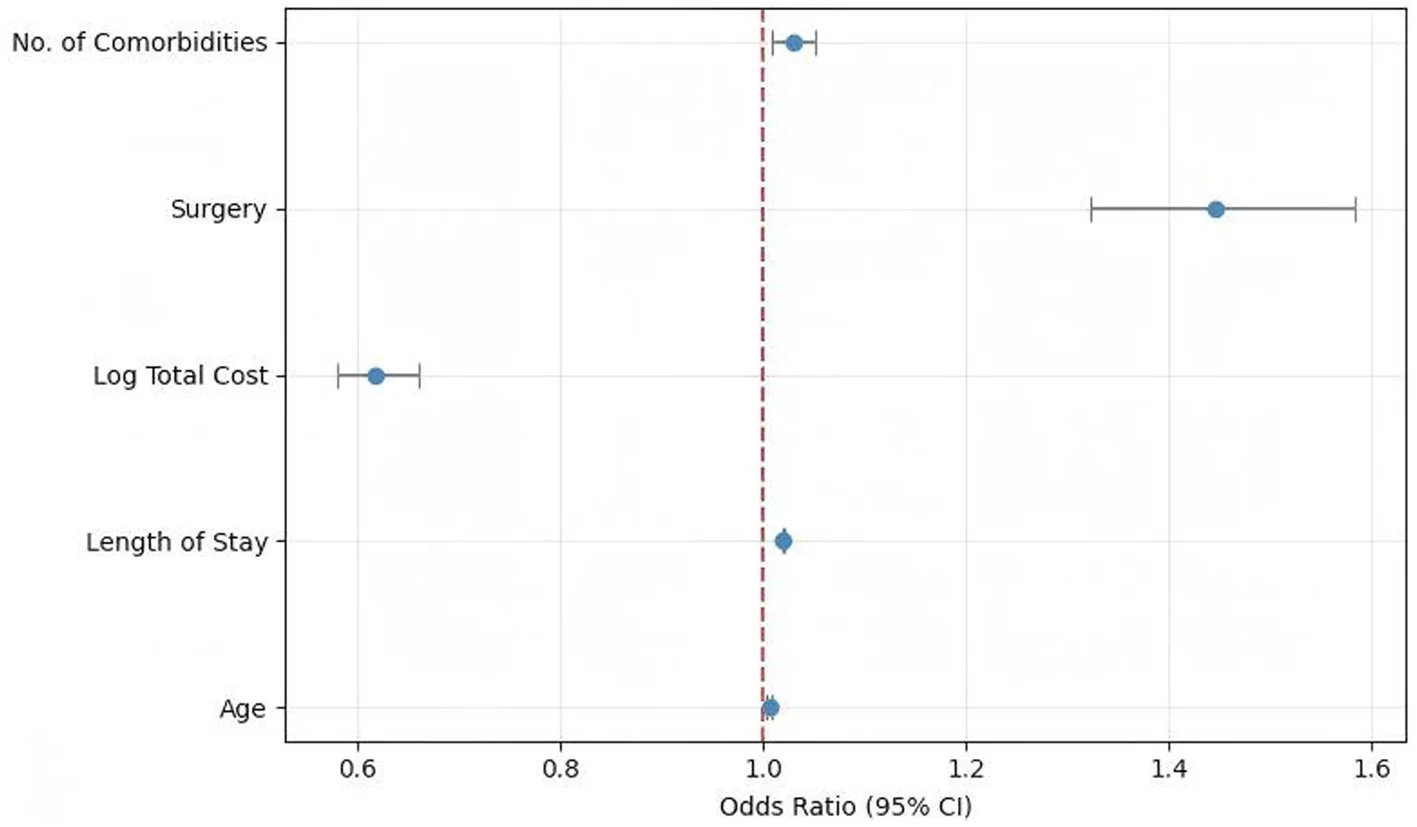
Forest plot of the 15-day model.

Figure 1 presents the forest plot of core variables in the 15-day readmission prediction model. The ORs for LOS (OR = 1.020), age (OR = 1.006), surgery (OR = 1.447), and number of secondary diagnoses (OR = 1.030) were greater than 1, suggesting higher odds of readmission; the OR for log-transformed total cost (OR = 0.619) was less than 1, indicating lower odds of readmission.

### 3.2 30-day readmission prediction model

The overall 30-day readmission rate was 10.92% (6,297/57,677). Results for the 30-day readmission prediction model are presented in Table 5. The direction of associations was in line with those observed in the 15-day model, but the magnitudes were generally smaller.

**Table 5:** Multivariable logistic regression for 30-day readmission.

Variable	OR	95% CI	P value
Age (per year)	1.005	1.003–1.007	<0.001
Length of stay (per day)	1.018	1.017–1.019	<0.001
Total cost (per log unit)	0.657	0.619–0.698	<0.001
Surgery (reference: no surgery)	1.435	1.320–1.560	<0.001
Number of secondary diagnoses (per unit)	1.037	1.018–1.056	<0.001
Year of discharge (reference: 2021)			
2022	1.838	1.535–2.200	<0.001
2023	12.436	10.573–14.628	<0.001
2024	13.150	11.140–15.524	<0.001
2025	6.104	5.110–7.290	<0.001
Primary diagnosis code	-	-	<0.001

Each 1-year increase in age was associated with a 0.45% increase in the odds of readmission (OR = 1.005; 95% CI: 1.003–1.007; *p* < 0.001). Each additional day of LOS was associated with a 1.81% increase in 30-day readmission odds (OR = 1.018; 95% CI: 1.017–1.019; *p* < 0.001). A 1-unit increase in the natural logarithm of total cost (corresponding to a 2.72-fold increase in cost) was associated with a 34.31% reduction in readmission odds (OR = 0.657; 95% CI: 0.619–0.698; *p* < 0.001). Surgery was associated with significantly higher odds of 30-day readmission (OR = 1.435; 95% CI: 1.320–1.560; *p* < 0.001). Each additional secondary diagnosis was associated with a 3.68% increase in odds (OR = 1.037; 95% CI: 1.018–1.056; *p* < 0.001).

Using 2021 as the reference year, the odds of readmission were significantly higher in 2022 (OR = 1.838; 95% CI: 1.535–2.200; *p* < 0.001), increased sharply to a peak in 2023 (OR = 12.436; 95% CI: 10.573–14.628; *p* < 0.001) and 2024 (OR = 13.150; 95% CI: 11.140–15.524; *p* < 0.001), and decreased in 2025 while remaining high (OR = 6.104; 95% CI: 5.110–7.290; *p* < 0.001).

As an overall categorical predictor, primary diagnosis code was significantly associated with readmission odds (overall *p* < 0.001).

**Figure 2:**
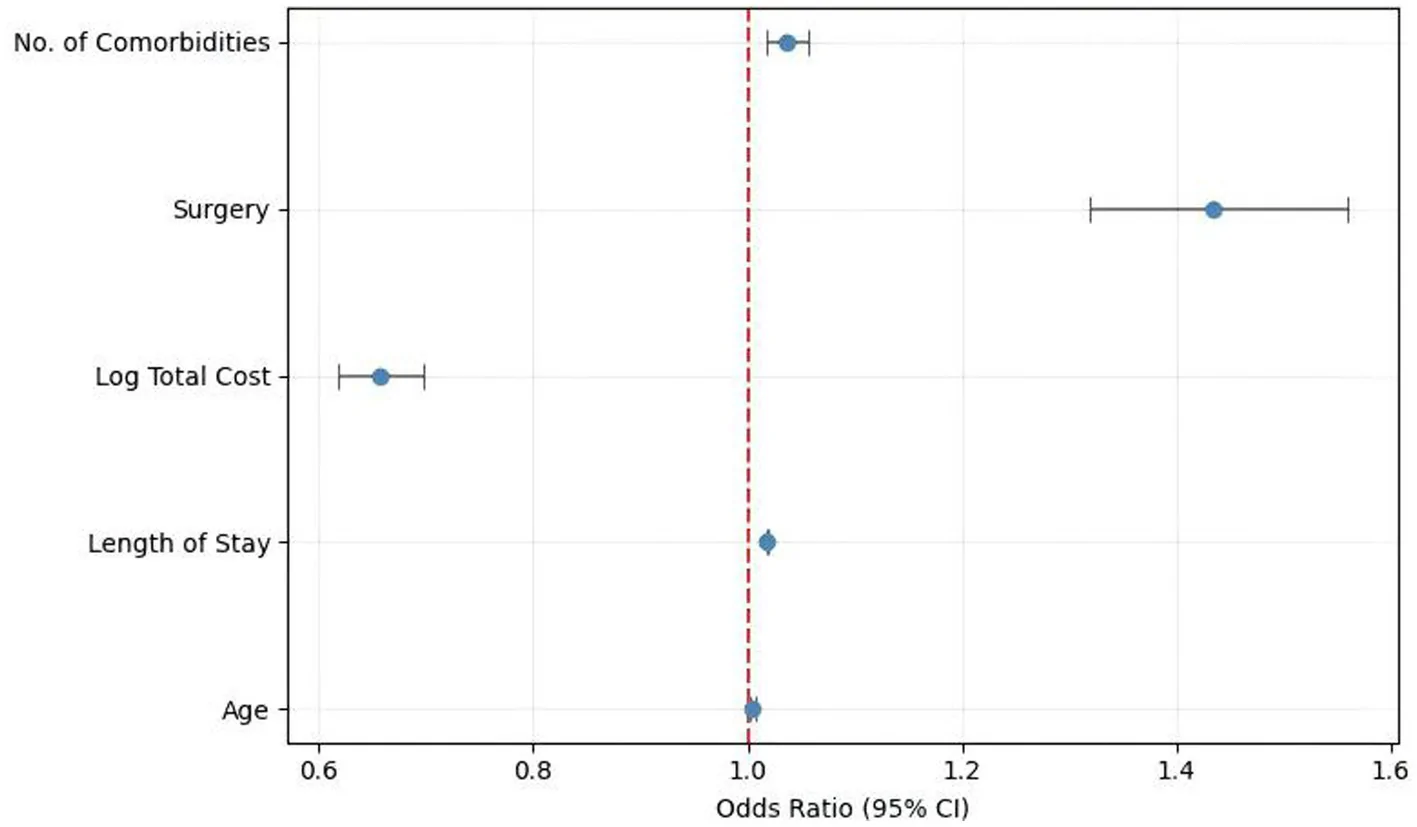
Forest plot of the 30-day model.

Figure 2 presents the forest plot of core variables in the 30-day readmission prediction model. Compared with the 15-day model, the association magnitudes for LOS (OR = 1.018) and age (OR = 1.005) were slightly smaller. The association for surgery was comparable between the two models (OR = 1.435 vs. 1.447), consistent with the interpretation that surgical status is a consistent predictor of readmission across both time windows.

In unadjusted comparisons, the median LOS in the readmission group (15-day model: 91 days; 30-day model: 90 days) was significantly longer than that in the non-readmission group (20 days). Nevertheless, in the fully adjusted multivariable models, the positive association between LOS and readmission odds remained (15-day model: OR = 1.020 per day; *p* < 0.001; 30-day model: OR = 1.018 per day; *p* < 0.001). These results imply that, within clinical pathways defined by a given diagnosis code, longer hospitalization may reflect greater clinical instability, treatment complexity, or unresolved disease, thereby being associated with a higher likelihood of early readmission.

To evaluate the linearity assumption for LOS, a sensitivity analysis was performed by categorizing LOS into quartiles and re-estimating the models. The overall association between LOS quartiles and readmission risk remained statistically significant (overall *p* < 0.001). Compared with the reference quartile, the highest LOS quartile showed a statistically significant association with readmission risk, whereas the middle quartiles exhibited inconsistent directional patterns. These results support the robustness of including LOS as a continuous predictor in the main models.

### 3.3 Risk heterogeneity between diagnosis codes

Primary diagnosis code was statistically associated with readmission odds as an overall categorical predictor, indicating considerable heterogeneity in readmission risk across diagnosis codes. To illustrate the degree of heterogeneity across primary diagnosis code, the highest-risk primary diagnosis codes in the 15-day model are presented in Table 6, and the highest-risk primary diagnosis codes in the 30-day model are presented in Table 7. Compared with the reference category, the highest-risk primary diagnosis codes in the 15-day model showed more than a 41-fold difference in adjusted odds. The highest-risk code was F71(OR = 42.559,95% CI: 5.501–329.244), which mainly covered patients with moderate intellectual disability requiring inpatient treatment. The elevated readmission risk associated with psychiatric diagnosis codes such as F71, F70, F72, and F20 may reflect both the clinical characteristics inherent to these conditions and differences in discharge preparedness incentives under the respective payment modalities. In the 30-day model, the highest-risk primary diagnosis codes showed smaller association magnitudes, with F71(OR = 10.43, 95% CI: 3.33–32.62) remaining the highest-risk code. These results indicate that 15-day readmission is more sensitive to risk differences at the DIP-code level and highlight structural heterogeneity in readmission risk across clinical pathways.

**Table 6:** Selected major primary diagnosis codes with highest adjusted odds of 15-day readmission.

Primary diagnosis code	OR	95% CI	*P*-value
F71	42.559	5.501–329.244	<0.001
F72	28.838	3.800–218.831	0.001
F70	28.420	3.6840–219.247	0.001
F20	19.822	2.733–143.797	0.003
F79	17.757	2.388–132.053	0.005

**Table 7:** Selected major primary diagnosis codes with highest adjusted odds of 30-day readmission.

Primary diagnosis code	OR	95% CI	*P*-value
F71	10.426	3.333–32.616	<0.001
F72	7.363	2.437–22.242	<0.001
F70	6.594	2.116–20.547	0.001
G82	6.261	1.944–20.168	0.002
F20	5.067	1.821–14.100	0.002

### 3.4 Model discrimination

The logistic regression models achieved good discrimination for readmission prediction.

**Figure 3:**
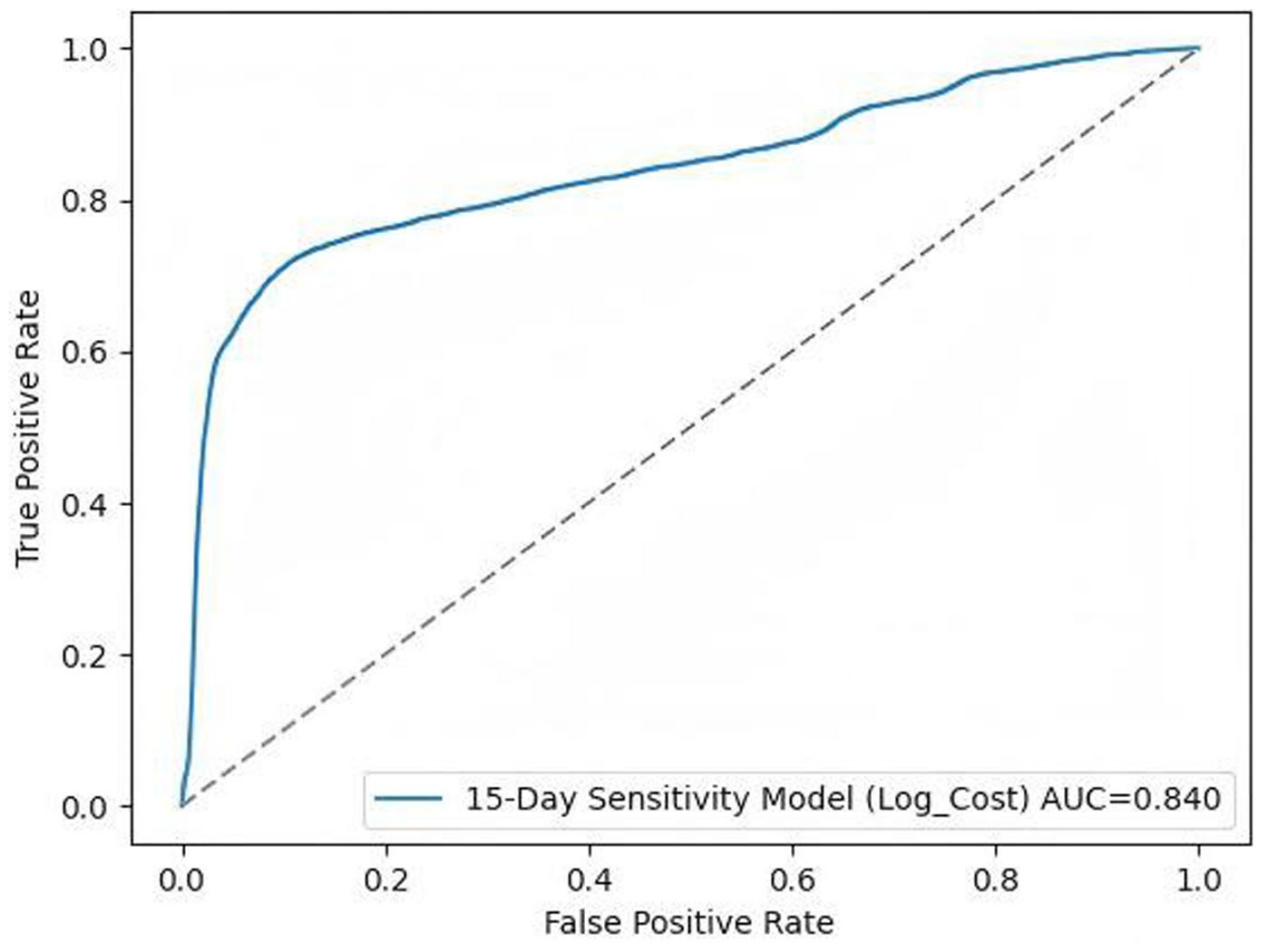
ROC curve of the 15-day readmission prediction model.

For the 15-day readmission prediction model, the AUC was 0.840 (95% CI: 0.828–0.843), as presented in Figure 3, reflecting good predictive accuracy. The optimal probability threshold derived from the Youden index was 0.1977, with a sensitivity of 0.7182 and a specificity of 0.8933. Thus, the model effectively discriminated between patients readmitted within 15 days and those who were not. Bootstrap internal validation yielded an optimism-corrected AUC of 0.836 and optimism of 0.004, further supporting model stability and generalizability.

**Figure 4:**
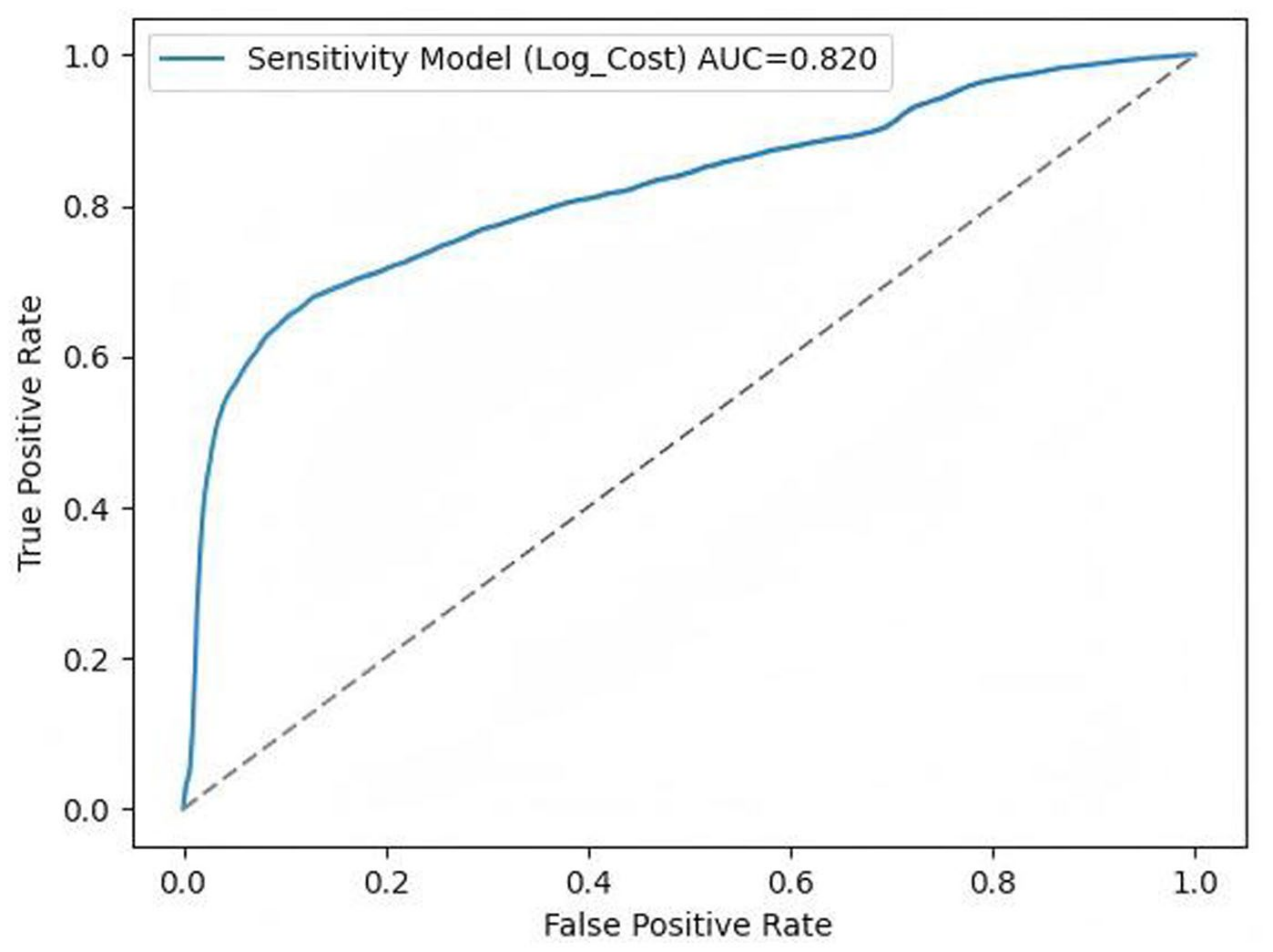
ROC curve of the 30-day readmission prediction model.

For the 30-day readmission prediction model, the AUC was 0.820 (95% CI: 0.810–0.825), as shown in Figure 4, which was lower than that of the 15-day model and indicated weaker discrimination for 30-day readmission than for 15-day readmission. The optimal probability threshold derived from the Youden index was 0.1886, with a sensitivity of 0.6786 and a specificity of 0.8718. These results indicate that the model was reasonably effective in distinguishing patients readmitted within 30 days from those who were not. Bootstrap internal validation showed an optimism-corrected AUC of 0.817 and optimism of 0.003, which was nearly identical to the apparent AUC, demonstrating no obvious overfitting and stable internal performance.

The AUC of the 15-day model (0.840) exceeded that of the 30-day model (0.820), corroborating previous evidence that proximal readmissions are more predictable than distal readmissions. These findings indicate that 15-day readmission is more sensitive to inpatient care quality and discharge preparation quality, whereas 30-day readmission may be affected by a broader set of outpatient and post-discharge factors.

### 3.5 External test

To further evaluate the predictive stability of the model across different temporal periods, temporal external validation was performed in this study. The hospitalization data from 2024 to 2025 were used as an independent validation cohort. The established 15-day and 30-day readmission prediction models were applied to the validation cohort without re-estimating model parameters. Model performance was evaluated using AUC and the Brier score. The temporal validation cohort included 22,079 patients, including 14,596 patients discharged in 2024 and 7,483 patients discharged in 2025. The results showed that the models maintained good predictive performance in the temporal external validation cohort, with an AUC of 0.8102 and a Brier score of 0.1361, indicating good discrimination and calibration performance.

### 3.6 Model calibration

**Figure 5:**
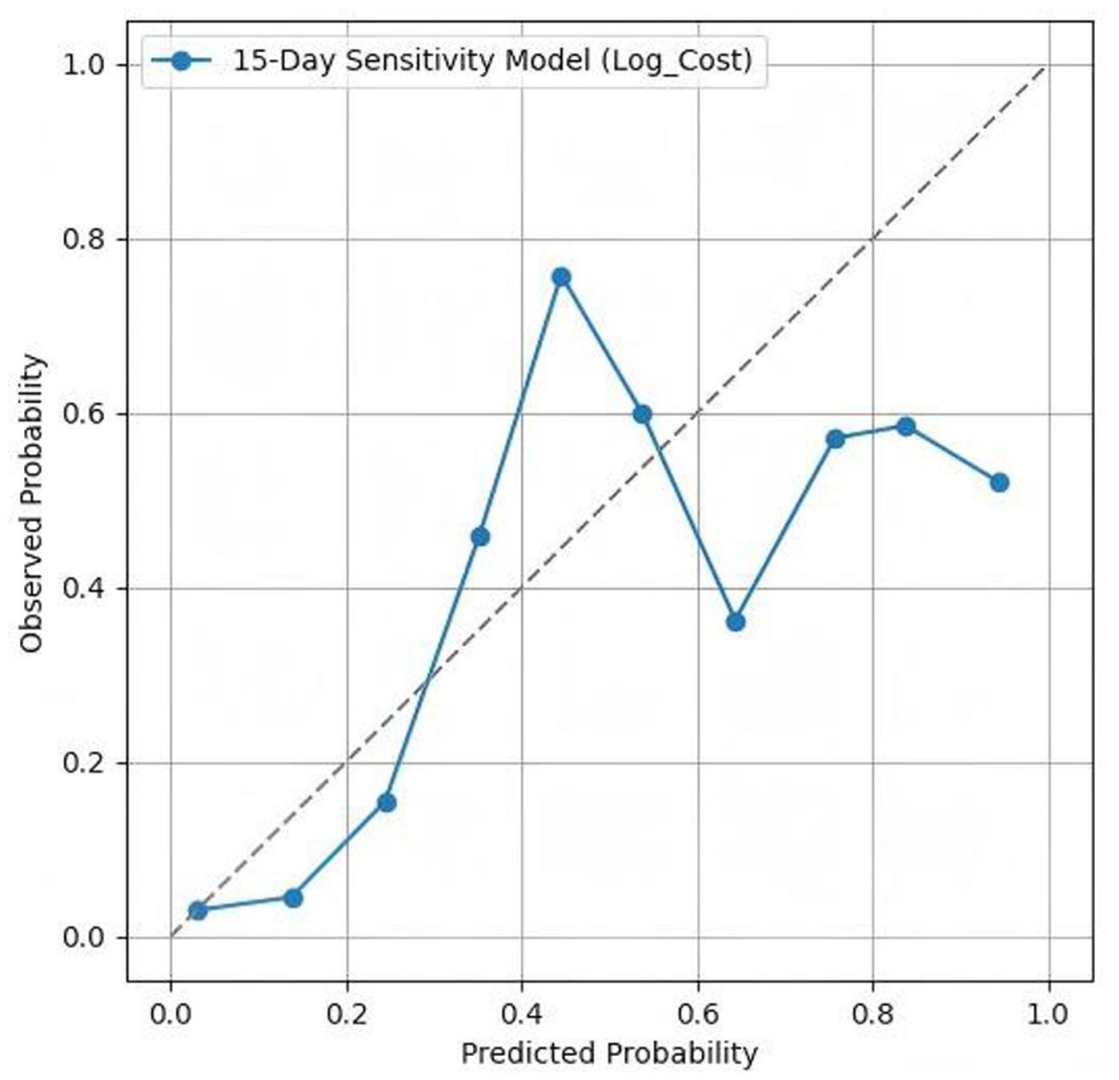
Calibration plot comparing predicted vs. observed 15-day readmission risk.

**Figure 6:**
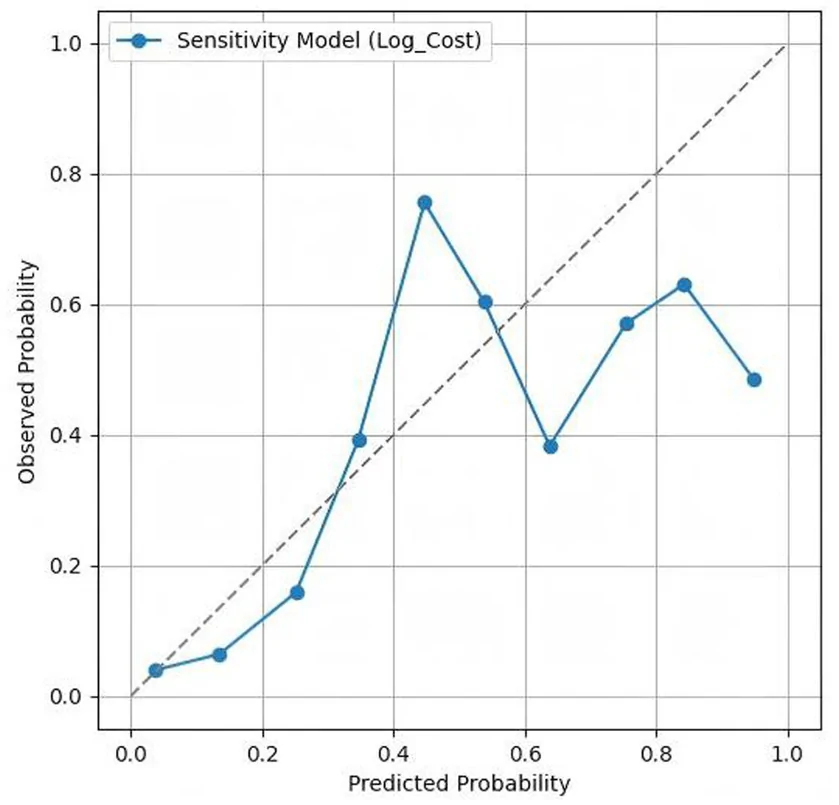
Calibration plot comparing predicted vs. observed 30-day readmission risk.

Figure 5 shows the calibration plot of the 15-day model, and Figure 6 shows the calibration plot of the 30-day model. In terms of overall accuracy, the Brier score was 0.0620 for the 15-day model and 0.0735 for the 30-day model. Both Brier scores were below 0.10, indicating good overall accuracy of predicted probabilities. The lower Brier score for the 15-day model (0.0620) indicates higher overall accuracy of predicted probabilities in the 15-day model. This finding further corroborates the conclusion that proximal readmission is more predictable than distal readmission.

### 3.7 Risk stratification

**Figure 7:**
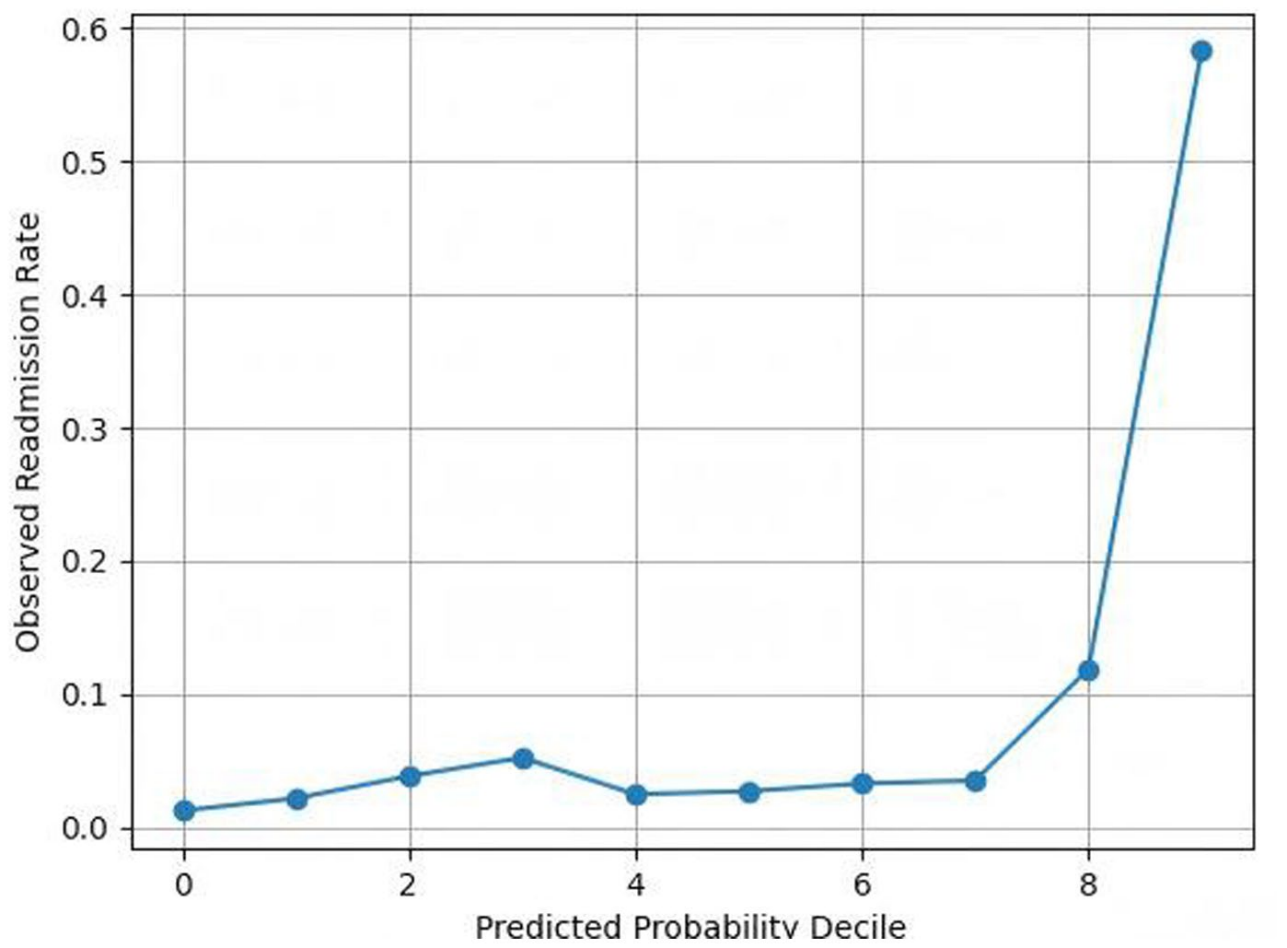
Risk stratification plot of the 15-day readmission prediction model.

**Figure 8:**
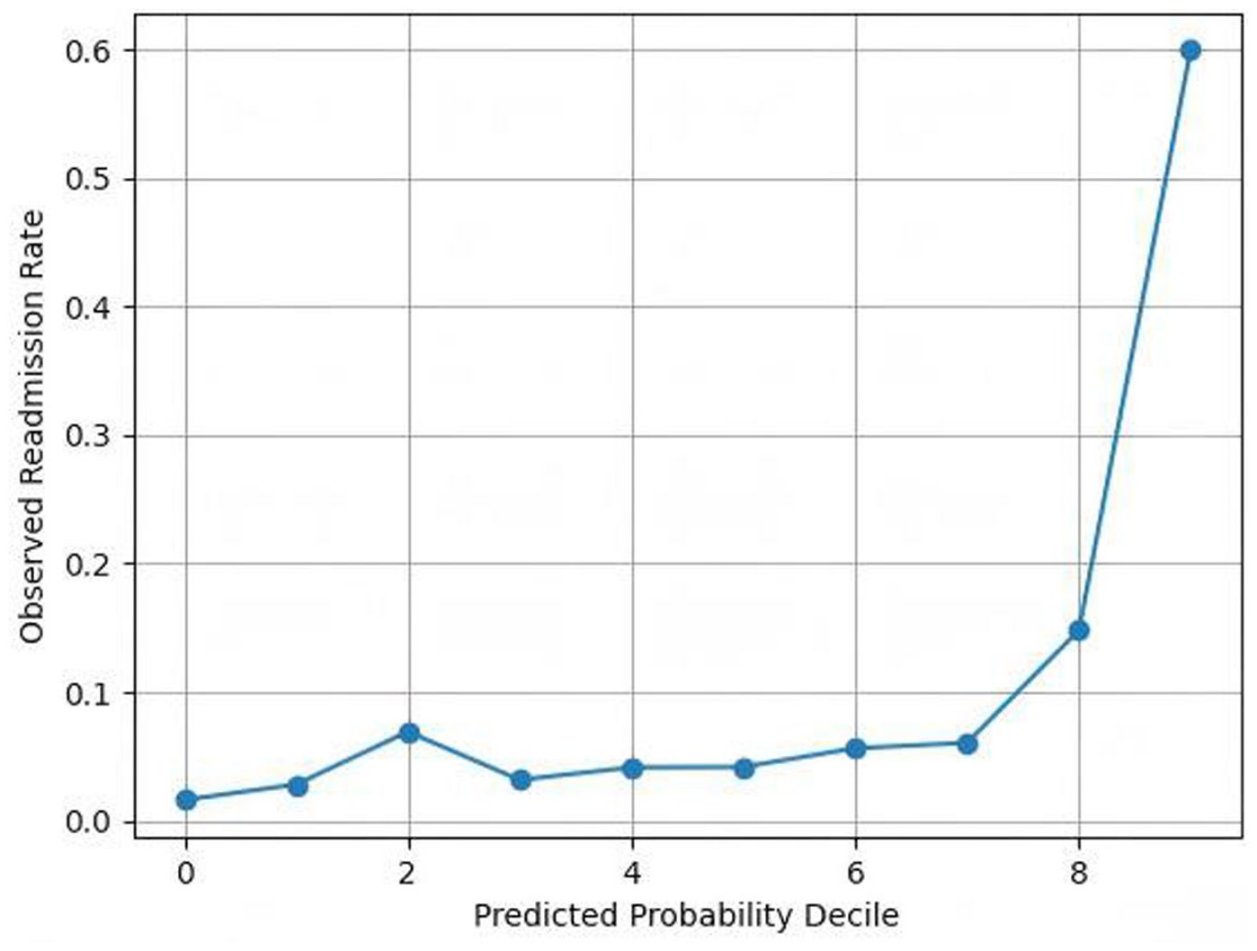
Risk stratification plot of the 30-day readmission prediction model.

Figure 7 presents the observed 15-day readmission rates stratified by deciles of predicted risk. The observed readmission rate increased from 1.27% in the lowest-risk decile to 58.27% in the highest-risk decile, with a clear gradient (lowest-risk decile, 1.27%; eighth decile, 11.89%; highest-risk decile, 58.27%). Figure 8 presents the observed 30-day readmission rates stratified by deciles of predicted risk. The observed readmission rate increased from 1.60% in the lowest-risk decile to 59.99% in the highest-risk decile, also with a clear gradient. Both models demonstrated good risk stratification ability, supporting their potential use in identifying high-risk patients and guiding targeted interventions.

## 4 Discussion

### 4.1 Principal findings and clinical implications

In this study, the 15-day and 30-day readmission rates were 9.47 and 10.92%, respectively, which were broadly consistent with international benchmarks for adult populations with mixed conditions (27). The present study identified distinct association patterns linking LOS, hospitalization costs, and surgical status with odds of readmission. These patterns may reflect the interplay of patient case mix, disease severity, and healthcare service process characteristics in shaping odds of readmission. After adjustment for age, comorbidities, diagnosis codes, and other covariates, length of stay remained positively associated with odds of readmission, although the effect size was relatively modest (15-day model: OR = 1.020 per day; 30-day model: OR = 1.018 per day). This finding suggests that patients with longer hospital stays tend to present with more complex disease states, higher treatment demands, and more concurrent complications; prolonged LOS may therefore primarily reflect clinical complexity rather than inadequate inpatient care rather than inadequate inpatient care itself. Previous studies have indicated that case-mix characteristics—including functional status, cognitive capacity, and comorbidity burden—are strongly associated with odds of readmission, and that conventional clinical indicators often fail to fully capture these dimensions (28) Accordingly, in the context of multi-payment reform, merely reducing length of stay is unlikely to lower odds of readmission; greater emphasis should be placed on individualized management of high-risk patients and post-discharge continuity of care.

In contrast to LOS, hospitalization costs (log-transformed) emerged as a protective factor for odds of readmission in the multivariable model. This result may reflect the change in what the cost variable represents after multivariable adjustment. When LOS, surgical status, disease type, and comorbidities are simultaneously included in the model, the cost variable predominantly reflects differences in the intensity of medical resource inputs within comparable disease burden and treatment contexts. Prior readmission prediction studies have noted that adequate covariate adjustment is essential for accurately interpreting the association between medical costs and readmission, and that some variables may exhibit a reversal in the direction of association after adjustment (23). Furthermore, disease severity and clinical complexity may be important factors shaping the relationship between costs and readmission. Patients with more severe conditions generally require more medical resources and are also at higher risk of readmission, yet conventional diagnosis codes and clinical indicators often fail to fully capture differences in functional status, complication burden, and treatment complexity (28). Therefore, after adjusting for key clinical characteristics, higher hospitalization costs may primarily reflect more thorough diagnostic and therapeutic input, more comprehensive treatment processes, and more adequate discharge preparation—factors that are, in turn, associated with lower short-term odds of readmission. Similar studies have also found that higher hospital spending intensity was associated with lower readmission rates, suggesting that reasonable resource input may be linked to improved quality of care (29). Meanwhile, the present study encompassed both DIP payment and per-diem payment modalities, and medical resource allocation and service behaviors may differ across payment mechanisms. Prior research has shown that payment incentives in mixed payment systems may shape healthcare service delivery patterns and, consequently, quality-of-care outcomes (9). In the context of payment reform, merely reducing medical costs does not necessarily improve patient outcomes, and excessive cost containment may compromise the quality of care (30). Thus, the protective association observed for costs more likely reflects the combined influence of resource inputs, case management quality, and payment mechanisms, rather than the direct protective effect of costs themselves. Surgery was identified as a risk factor for readmission in both time-window models, with the stronger association observed in the 15-day model than in the 30-day model, suggesting that surgery-related risk may be concentrated in the early post-discharge period. Surgical patients generally face higher perioperative risk, and complications such as postoperative infection, bleeding, and inadequate functional recovery may represent important factors in early readmission. Prior studies have indicated that postoperative readmissions are primarily associated with new surgery-related complications arising after discharge, and that postoperative complications are important predictors of readmission (31, 32). In addition, differences in hospital surgical quality and perioperative management capacity may also be associated with postoperative odds of readmission (33). Therefore, the association between surgical status and higher odds of readmission observed in the present study does not imply that surgery per se is directly responsible for readmission; rather, it may reflect greater disease complexity and higher postoperative management needs among patients who undergo surgery.

The highest-risk primary diagnosis codes were concentrated in categories such as intellectual disability (F70-F72), paraplegia (G82), and schizophrenia (F20). Under the current multi-payment reform, psychiatric diagnosis codes such as F70–F72 and F20 are predominantly settled through per-diem payment at a fixed daily rate. The elevated readmission risk in this patient group may be explained at two levels. First, the chronic features inherent to these conditions, including cognitive impairment, limited self-care capacity, insufficient family caregiving resources, and poor medication adherence—in themselves constitute risk factors for readmission (34). Second, the per-diem payment mechanism, which relies on a fixed daily settlement, may weaken the constraints on discharge preparedness quality and transitional care within the incentive structure, and may be further associated with elevated readmission rates (35). International evidence has also confirmed this pattern. In a cohort study of adults with intellectual disabilities in the United Kingdom, Sheehan et al. found that schizophrenia-spectrum disorders were among the strongest predictors of admission and readmission (34). Rast et al., using the US Nationwide Readmissions Database, showed that adults with intellectual disabilities had significantly higher 30-day readmission rates than those without intellectual disabilities (36). This pattern aligns with findings from the United Kingdom and the United States, suggesting that structural differences in readmission risk are a common feature of modern hospital systems. The present study also found that, compared with 2021, patients discharged in 2023 and 2024 exhibited markedly higher odds of readmission. This temporal trend may reflect the combined influence of multiple factors, including the phased impact of multi-payment reform implementation, adjustments in provider behavior, changes in patient case mix, and shifts in the external healthcare environment. First, the effects of multi-payment reform have unfolded in stages, with their effects evolving over time. The DIP payment reform has undergone policy adjustments, refinement of payment rules, and gradual adaptation by healthcare institutions during its implementation, and its effects on care quality and efficiency may depend on local implementation conditions and supporting management measures. Prior studies have shown that the role of payment reform in cost containment and quality improvement varies across regions, and that reform outcomes may depend on payment rule design, the adequacy of management measures, and the adaptation process of healthcare institutions (37, 38). Thus, the year effects observed for 2023 and 2024 may reflect the phased adjustments in clinical practices and management models of healthcare institutions during the advancement of payment reform, rather than simply representing the decline in care quality. Furthermore, dynamic changes in patient case mix may also shape the interpretation of the year variable. Prior studies have found that payment reform may alter the patient mix and service delivery patterns of healthcare institutions, and that some institutions may adjust their admission strategies in response to changes in the payment system, with corresponding changes in patient disease severity and treatment complexity (39). If the clinical variables included in the model fail to fully capture differences in disease severity, comorbidity burden, and treatment intensity, these unobserved factors may be further absorbed by the year variable, manifesting as higher odds of readmission. Conversely, as the public health environment evolved and healthcare services gradually returned to normal operations, patient care-seeking patterns and case mix may have shifted, further shaping odds of readmission during 2023 and 2024. Prior research have suggested that as health systems returned to normal operations as healthcare services resumed routine operations following major public health disruptions, inpatient volumes, case mix, and resource utilization may have shifted, inpatient volumes, disease spectra, and medical resource utilization patterns may shift, and these changes may further shape healthcare utilization patterns and temporal trends in odds of readmission (40). In summary, the markedly higher ORs for 2023 and 2024 are likely the product of multiple converging factors, including the advancement of health insurance payment reform, adaptive behaviors of healthcare institutions, changes in patient case mix, and shifts in the external healthcare environment. As the long-term effects of prospective payment systems on quality indicators, including readmission, remain subject to some uncertainty, further exploration of the underlying mechanisms using multicenter data and longitudinal study designs is warranted (41).

In the comparison between time windows, the 15-day model showed better discrimination (AUC, 0.840) and calibration (Brier score, 0.0620) than the 30-day model (AUC, 0.820; Brier score, 0.0735). This difference may reflect the distinct risk mechanisms captured by readmission events across the two-time windows. Early readmissions are typically more closely linked to inpatient treatment processes, discharge preparation quality, care transitions, and postoperative complications, whereas later readmissions may be more strongly shaped by disease progression, long-term health management, and out-of-hospital factors. Consequently, readmission events within the 15-day window may contain a higher proportion of risk signals explainable by clinical information available during the index hospitalization, yielding higher predictive performance. Prior studies have found that early readmission are more preventable and that hospitals play a more prominent role in reducing early odds of readmission; in addition, predictive model performance differs across time windows, with proximal readmission models generally exhibiting better discrimination (17, 18) Overall, the differences in predictor associations between the 15-day and 30-day models reflect the distinct risk mechanisms operating across the two-time windows: 15-day readmissions are more strongly driven by factors related to inpatient care and discharge management, whereas 30-day readmissions may be further shaped by long-term disease management and out-of-hospital factors. This dual-window design provides differentiated tools to support stratified quality assessment in the context of multi-payment reform. Age, length of stay, surgical status, and number of secondary diagnoses were independent predictors of early readmission, consistent with patterns documented across multiple OECD health systems (42). Thus, in the context of multi-payment reform, adopting a dual time-window prediction strategy may facilitate more precise identification of odds of readmissions at different stages. This finding has policy-tool value for coordinated tripartite medical reform. For medical insurance, 15-day readmission may be used as a sensitive indicator for medical quality assessment, whereas 30-day readmission may serve as a supplementary indicator for performance evaluation. For healthcare delivery, the high sensitivity of the 15-day window enables rapid identification of in-hospital management problems, while the 30-day window may help identify opportunities for improvement in post-discharge follow-up and chronic disease management. For pharmaceutical management, 15-day readmission can serve as an indicator for evaluating the quality of discharge medication plans and the effectiveness of medication counseling. Accordingly, the dual-window design provides differentiated monitoring targets and stratified intervention nodes for coordinated tripartite medical reform.

### 4.2 Comparison with existing international evidence

International experience has shown that incorporating readmission indicators into healthcare payment systems is not merely the matter of simply establishing penalty mechanisms; rather, it requires a comprehensive governance framework that integrates risk adjustment, readmission management, and payment incentives. Countries have gradually recognized during payment reform that odds of readmission is shaped by multiple factors—including disease severity, functional status, social support, and continuity of care—and that evaluating hospital quality solely based on readmission rates may yield unfair incentives. A robust risk adjustment mechanism therefore constitutes the foundation for achieving both fairness and effectiveness in readmission management.

With respect to risk adjustment, experience with the U.S. Medicare Hospital Readmissions Reduction Program (HRRP) has demonstrated that incorporating readmission indicators into payment evaluation requires thorough consideration of patient clinical complexity and social risk factors. Prior research has indicated that inadequate risk adjustment may expose hospitals serving disproportionately more vulnerable patients and complex cases to unreasonable penalties, thereby compromising payment equity (43). Japan’s DPC/PDPS payment system similarly emphasizes differentiated management tailored to disease type and healthcare needs, using group-based payment and adjustment mechanisms to mitigate the adverse effects of uniform payment standards on specialized conditions (44). The present study found that patients classified under diagnosis codes for intellectual disability (F70–F72), paraplegia (G82), and schizophrenia (F20) were at higher odds of readmission, suggesting that, if readmission indicators are to be further applied to quality evaluation or payment incentives within China’s multi-payment environment, disease type and patient risk heterogeneity should be fully accounted for and case-mix-based risk adjustment strategies should be established.

Regarding readmission management, international practice has progressively shifted from a narrow focus on readmission outcomes toward strengthening post-discharge continuity of care and early risk intervention. France’s PRADO program, which introduced care coordinators during hospitalization, developed post-discharge care plans, and strengthened the interface between hospitals and primary care institutions, was associated with lower readmission rates among high-risk patients (45). The Dutch cross-boundary care model, which established collaborative management pathways spanning hospitals, communities, and households to deliver early post-discharge follow-up, health education, and medication management for patients with chronic conditions, was linked to reduced hospitalization needs (46). These experiences suggest that the key to odds of readmission management is not simply controlling the use of medical resources; rather, it lies in providing continuous and targeted health management support during the critical post-discharge window. The present study found that the 15-day readmission model demonstrated superior predictive performance relative to the 30-day model, suggesting that odds of readmission is more predictable during the early post-discharge period. This finding also provides a basis for establishing the readmission management model centered on early identification and proactive intervention in the context of China’s payment reform.

With respect to payment incentives, different countries have explored implementation pathways that combine financial incentives with quality improvement. In the United States, the HRRP linked readmission indicators to payment adjustments to encourage hospitals to prioritize readmission reduction; however, studies have found that the long-term effects of financial penalties alone on healthcare quality improvement remain uncertain, and that such mechanisms need to be combined with clinical quality improvement measures and organizational management support (47). France has placed greater emphasis on promoting institutional improvement through public reporting of quality indicators and regional coordination, rather than through direct financial penalties (48). Prior research has also indicated that, in the context of payment reform, the excessive emphasis on cost containment may be associated with reduced quality of care, and that payment incentives need to strike the balance between cost control and patient safety (30). In China, the implementation of multi-payment modalities, including DIP and per-diem payment, has been accompanied by adjustments in healthcare service delivery models, yet quality indicators such as readmission have not been fully integrated into the payment evaluation system. Meanwhile, China’s health insurance reform has focused not only on controlling healthcare expenditures and improving the efficiency of health insurance fund utilization, but also on promoting equitable allocation of healthcare resources and strengthening health security through institutional integration. Previous research has shown that the integration of urban and rural health insurance schemes has contributed to improving equity in health insurance coverage and narrowing the urban–rural income gap, highlighting the important role of health insurance policies in promoting equitable resource allocation and social equity (49). The dual time-window readmission prediction model constructed in the present study, which quantifies risk variation across different time windows and diagnosis categories, provides the empirical foundation for developing a coordinated framework that integrates risk-based readmission monitoring with payment design and quality improvement.

In summary, international experience suggests that readmission management requires a shift from outcome-based evaluation toward a precision management model grounded in risk adjustment, supported by early intervention and appropriately designed payment incentives to facilitate sustained improvement in care quality. The dual time-window prediction model constructed in the present study, based on real-world inpatient data, provides a key reference for strengthening odds of readmission surveillance systems, refining payment modality design, and advancing the coordinated development of quality of care and cost containment in the context of China’s multi-payment reform.

### 4.3 Policy insights for coordinated tripartite medical reform in multi-payment reform

The findings of this study indicate that readmission risk is not merely a quality outcome indicator; it also provides a quantifiable basis for decision-making on how cost containment and quality assurance can be reconciled under multi-payment reform. The findings support the notion that 15-day and 30-day readmission risks under the multi-payment reform had distinct risk factor profiles and temporal sensitivities, providing an empirical basis for developing a tiered and stratified quality monitoring system. Building on these results, the policy implications of the findings for payment design, hospital management, and pharmaceutical resource allocation are examined from the perspective of coordinated tripartite medical reform.

In terms of medical insurance payment and management, a dual-window readmission risk monitoring system should be established at the primary diagnosis code level. For high-risk codes such as F71, F72, and G82, risk-adjustment increments should be applied in score calculation to prevent medical institutions that admit such patients from bearing disproportionate quality penalties. The prediction model developed in this study may also be used to provide appropriate exemption or compensation for high-risk readmission events within the 15-day window during payment settlement, thereby avoiding adverse selection leading to “low price and low quality.” The dual-window design may help medical insurance authorities ensure quality while containing costs, aligning the efficiency and public-interest functions of pricing instruments. At the level of hospital management, the prediction model should be used for risk stratification before discharge. The risk stratification plot in this study showed that the observed readmission rate exceeded 58% in the highest-risk decile, whereas it was below 2% in the lowest-risk group. This large gradient provides ample opportunity for stratified clinical intervention. Enhanced transitional care should be implemented for high-risk patients, including post-discharge telephone follow-up, medication reconciliation, and home visits. Meanwhile, the 15-day readmission rate should be used as a sensitive indicator for internal quality improvement to rapidly identify in-hospital management problems, whereas the 30-day readmission rate should be used as an indicator for validating the performance of chronic disease management and post-discharge follow-up. The positive association between LOS and readmission risk suggests that LOS is not merely an efficiency indicator but also a marker of case complexity; therefore, medical institutions should not shorten LOS indiscriminately for all patients for the purpose of cost containment. In pharmaceutical management, differentiated strategies should be adopted for the two-time windows. The 15-day readmission window is more sensitive to adverse drug reactions and may serve as a short-term evaluation indicator for the quality of discharge medication plans. For high-risk patients with intellectual disability, schizophrenia, and related conditions, medication regimens should be optimized, with pharmacist involvement in refining discharge prescriptions. For the 30-day window, pharmacists should focus on long-term medication adherence and participate in post-discharge follow-up for patients with high-risk codes to identify and address medication-related problems in a timely manner. From the perspective of overall policy design, firstly, with respect to integration into inpatient management systems, the readmission prediction model could be embedded within hospital electronic health record (EHR) systems and hospital information systems (HIS) to dynamically calculate odds of readmission using clinical data continuously updated during the patient’s hospital stay, generating risk-stratified alerts on physician workstations or nursing management platforms. This approach would transform the conventional model of retrospective readmission evaluation into proactive risk identification during hospitalization, thereby supporting clinical decision-making and quality management. As public hospital governance continues to shift from expansion-oriented development toward quality improvement and refined management, information technologies have become increasingly important in optimizing hospital operations, enhancing healthcare quality, and supporting evidence-based management decisions (50). Therefore, integrating risk prediction models into hospital information management systems can not only facilitate dynamic identification of patient risks but also promote a transition from outcome-based evaluation to process-oriented quality management. Second, regarding real-time risk warning, the model could inform a tiered alert mechanism based on patient risk levels. For patients predicted to be at high risk, the clinical team could conduct early case reviews, with a focus on disease complexity, treatment response, and potential risk factors for readmission at discharge. In addition, given the finding that the 15-day model demonstrated superior predictive performance, hospitals could use short-term risk warning as a critical intervention window centered on inpatient care quality, perioperative management, and discharge transition, thereby identifying high-risk patients early and reducing the occurrence of avoidable short-term readmissions. Third, concerning the formulation of individualized discharge plans, model predictions could serve as an important basis for developing differentiated discharge strategies. For high-risk patients, hospitals could strengthen comprehensive pre-discharge assessments—including treatment efficacy evaluation, medication management guidance, functional recovery assessment, and family caregiving capacity analysis—and adjust follow-up frequency and management protocols according to the patient’s risk level. For populations at particularly high odds of readmission, such as those with mental disorders, functional limitations, or high chronic disease burdens, discharge preparation and referral coordination should be further strengthened to reduce readmissions arising from inadequate treatment continuity. Fourth, in supporting continuous care management, the prediction model could be integrated with out-of-hospital health management systems to facilitate information linkage among hospitals, primary care institutions, and patients. For high-risk patients, model-derived results could guide the implementation of early post-discharge follow-up, health education, medication monitoring, and remote health management interventions, extending care from in-hospital treatment to out-of-hospital management. Prior studies have indicated that coordinated care and continuous management during the post-discharge transition period were associated with improved continuity of care and lower odds of readmission. Therefore, combining risk prediction tools with the hierarchical medical system and continuous care frameworks may further enhance the efficiency of readmission management.

Overall, the dual time-window prediction model developed in the present study is applicable not only to odds of readmission assessment but also to providing an implementation pathway for healthcare institutions to conduct proactive quality management. Following further external validation and model calibration, the prediction model could be explored for integration into hospital quality control systems and payment reform evaluation frameworks. Through an integrated approach combining risk identification, targeted intervention, and continuous management, such integration may facilitate the optimization of medical resource allocation and the improvement of healthcare service quality.

## 5 Conclusion

This study constructed and validated dual time-window readmission prediction models based on 57,677 real-world inpatient records, establishing a new analytical framework for the precise identification of short-term odds of readmission. The performance of the 15-day and 30-day prediction models was compared, and differences in odds of readmission factors and predictive capacity across time windows were identified, suggesting that selecting an appropriate prediction window tailored to the phase of risk occurrence is important for improving risk identification accuracy. Associations between patient clinical characteristics, healthcare service utilization patterns, disease categories, and odds of readmission were further identified, offering real-world evidence that may support healthcare institutions in the early identification of high-risk patients, refinement of discharge management, and strengthening of continuous care delivery. In the context of multi-payment reform, these findings provide methodological support for healthcare quality monitoring and odds of readmission management.

However, this study has several limitations. First, the data were derived from hospital administrative records and lacked detailed stratification of comorbidities, physiological indicators, frailty status, and functional capacity, which may have introduced residual confounding. Second, readmission events were identified based on inpatient records from the same medical institution, and cross-institutional readmissions could not be captured; the actual readmission rate may therefore have been underestimated. Third, given the absence of reliable indicators in the administrative data for distinguishing planned from unplanned readmissions, all-cause same-hospital readmission within a defined post-discharge time window was used as the study outcome, rather than strictly defined unplanned readmission. A proportion of planned readmissions may consequently have been captured in the analysis, introducing some degree of overestimation of the readmission rate. Fourth, as a single-center retrospective study, external generalizability is limited; the data were sourced from a single large tertiary hospital, whose case mix and care delivery model may differ from those of lower-tier institutions, and the detailed implementation rules for payment modalities have not yet been fully standardized across regions. Model extrapolation therefore requires multicenter external validation. Fifth, individual-level information on payment modality was limited. Although both DIP and per-diem payment were utilized at the study hospital, the administrative data did not contain case-level fields specifying the settlement method applied; therefore, no direct comparison of readmission risk across payment types was performed.

Future research could be extended in the following directions. First, as the prediction models were constructed using single-center real-world inpatient data, multicenter external validation across different regions and types of healthcare institutions is still needed to further assess model stability and generalizability. Second, with the continued advancement of multi-payment reform, future studies could further explore the applicability of readmission prediction models across different payment environments, taking into account payment type and regional implementation characteristics—and refine risk adjustment strategies accordingly. Third, the present study relied primarily on clinical and administrative data from the index hospitalization for risk prediction; future research could further incorporate dynamic information on post-discharge healthcare utilization, primary care follow-up, and remote health monitoring to construct dynamic prediction models capable of continuously updating risk probabilities, thereby enhancing the capacity to predict long-term patient health outcomes. Finally, future studies could investigate pathways for integrating prediction models with clinical decision support systems and quality management frameworks, facilitating the translation of odds of readmission identification from a research tool into an instrument for healthcare quality improvement. In summary, this study constructed and validated dual time-window readmission prediction models based on real-world data, providing empirical evidence for odds of readmission surveillance and quality evaluation in the context of multi-payment reform. Multicenter validation, dynamic data integration, and clinical application evaluation are still needed to further enhance the generalizability and practical utility of the predictive tool, thereby supporting the coordinated advancement of healthcare quality improvement and payment reform.

## Data Availability

The original contributions presented in the study are included in the article/supplementary material, further inquiries can be directed to the corresponding author/s.
